# Laboratory-acquired Brucellosis

**DOI:** 10.3201/eid1010.040076

**Published:** 2004-10

**Authors:** Stephanie Noviello, Richard Gallo, Molly Kelly, Ronald J. Limberger, Karen DeAngelis, Louise Cain, Barbara Wallace, Nellie Dumas

**Affiliations:** * New York State Department of Health, Albany, New York, USA;; † Centers for Disease Control and Prevention, Atlanta, Georgia, USA;; ‡ Saint Agnes Hospital, White Plains, New York, USA

**Keywords:** brucellosis, laboratory-acquired, Gram stain, dispatch

## Abstract

We report two laboratory-acquired *Brucella melitensis* infections that were shown to be epidemiologically related. Blood culture isolates were initially misidentified because of variable Gram stain results, which led to misdiagnoses and subsequent laboratory exposures. Notifying laboratory personnel who unknowingly processed cultures from brucellosis patients is an important preventive measure.

The incidence of brucellosis may reach 200 per 100,000 population in some developing countries, but in the United States brucellosis is a rare disease (*1*). Over the past 50 years, effective control of brucellosis in animals and animal products in the United States has dramatically reduced the number of infections (*2*). Because of the rarity of cases, laboratory and medical personnel may not be familiar with *Brucella*, its clinical manifestations, and its laboratory characterization.

Transmission of brucellosis occurs from ingesting, directly contacting, or inhaling the organism. Exposures most commonly occur by eating contaminated animal products from disease-endemic areas (*1*). Other, less common routes include person-to-person transmission and accidental infection with live animal vaccines (*3**,**4*). *Brucella* species are classified as category B bioterrorism threat agents, so use as a bioweapon must also be considered as a potential source of exposure (*5*).

Laboratory workers are at risk when handling specimens containing *Brucella* species because of aerosol-generating procedures or accidents that may result in infection of blood or conjunctiva (*6*). In fact, brucellosis is one of the most common laboratory-acquired infections (*6**–**14*). To identify *Brucella* spp. from blood cultures, laboratory workers rely on the Gram stain as a preliminary test. *Brucella* organisms are gram-negative coccobacilli and may be present in high concentration in blood cultures drawn early in the disease process. If the Gram stain is misinterpreted, the organism may be misidentified, which would result in misdiagnosis and potential opportunities to expose laboratory personnel (*10*).

From 1993 to 2002, five *B. melitensis* and three *B. abortus* infections were reported in New York State, exclusive of New York City. Of the eight reported cases, two were laboratory-acquired, five resulted from ingesting unpasteurized milk products from countries with endemic disease, and one had an undetermined source of infection (New York State Department of Health [NYSDOH], unpub. data). We describe two cases of laboratory-acquired brucellosis in New York State that were not known to be related at the time of their diagnoses. Initial isolates from blood cultures of an index patient and two laboratory workers were incorrectly identified as contaminants, in part because of reporting of primary Gram stain results as gram-positive and gram-variable.

## Case Reports

In early November 2001, a 57-year-old female laboratory worker (laboratory worker 1) began experiencing nonspecific symptoms of malaise, vomiting, headache, lower leg cramping, anorexia, and fever. One week after onset of symptoms, she was evaluated for severe headaches at a local emergency room, where cerebrospinal fluid (CSF) and blood cultures were collected. The CSF culture was negative. From the blood culture, small, gram-positive bacilli were isolated and characterized as coryneform bacilli, which are usually interpreted as contaminants of unknown clinical importance. Despite multiple hospital admissions, the laboratory worker continued to have symptoms, but her condition remained undiagnosed. Approximately 5 weeks after symptom onset, colleagues from the hospital microbiology laboratory where she was employed (laboratory 1) drew her blood for culture again. After 5 days of incubation, gram-variable coccobacilli, later identified as *Brucella* spp., were isolated. Subculturing and biochemical tests were conducted in a class II biosafety cabinet. The *Brucella* serum agglutination test (SAT) was reactive (1:640) at the NYSDOH laboratory, Wadsworth Center. Laboratory worker 1 was initially treated with doxycycline and gentamicin, followed by doxycycline and rifampin, for 6 weeks of outpatient therapy. The isolate was later identified as *B. melitensis* by the Wadsworth Center and confirmed by the Centers for Disease Control and Prevention. The patient has not relapsed 18 months after completing treatment.

In a second incident in mid-January 2002, a 48-year-old woman had nocturnal temperature spikes to 40°C, chills, drenching sweats, and weight loss. Initially, she had a diagnosis of influenza and was treated with oseltamivir phosphate. Symptoms persisted, and uveitis developed. In early March 2002, a diffuse, erythematous rash appeared on the anterior aspect of both legs. A blood culture and serologic tests for Lyme disease, ehrlichiosis, and Rocky Mountain spotted fever (RMSF) were performed. From the blood culture, gram-positive cocci were isolated and identified as *Micrococcus* spp. by a commercial laboratory. RMSF titers were immunoglobulin (Ig) M-negative with a reactive IgG of 1:256. Her physician prescribed 3 weeks of doxycycline for RMSF, and the fevers resolved. Subsequently, she was referred to an infectious disease specialist, who found repeat RMSF titers unchanged, which made acute RMSF unlikely. Additional testing identified a reactive *Brucella* SAT (1:640). When interviewed by NYSDOH staff, the patient reported that she was a laboratory worker (laboratory worker 2) at laboratory 2. Her initial blood culture specimen, which had originally been identified as *Micrococcus*, was reassessed by the commercial laboratory. The commercial laboratory referred the original isolate to the Wadsworth Center, where the isolate was identified as *B. melitensis*.

No evidence of exposure to *Brucella* spp., other than through occupational exposure, was identified for either laboratory worker. They denied traveling outside of the United States, consuming imported or domestic unpasteurized dairy products, knowing ill family or friends who may have traveled, attending events with potentially contaminated foods, or handling farm or laboratory animals. Both laboratory workers denied any accidental contamination or spills in the laboratory during the 6 months before their respective illnesses. Site visits to laboratories 1 and 2 were conducted by NYSDOH. On the open bench at laboratory 1, blood cultures were routinely subcultured onto agar. A syringe was used to directly plate contents of the blood culture media bottle onto agar. A Gram stain was then performed with additional contents from the syringe. The stain was fixed in an incubator. Subsequent biochemical tests, including a catalase test, which may generate aerosols by introducing hydrogen peroxide to the specimen, were performed on the open laboratory bench. At laboratory 2, subculturing occurred in a similar manner, but it took place in a class II biosafety cabinet. However, biochemical tests were performed on the open laboratory bench when Gram stain results indicated that spores were not present. Laboratory workers 1 and 2 wore gloves when processing specimens, and both denied having dermatitis or skin lesions on their hands.

After the diagnosis of brucellosis in these two laboratory workers, serum samples from their co-workers from laboratories 1 and 2 were tested by *Brucella* SAT; samples from seven of eight co-workers were nonreactive (<1:20). A co-worker of laboratory worker 1 had an initial agglutination titer of 1:40 (indeterminate) and 1 month later had a repeat titer of <1:20; she denied having symptoms.

To determine the source of these laboratory workers' infections, each laboratory reviewed Gram stains of blood culture specimens processed within 3 to 6 months of symptom onset. Laboratory worker 2 reviewed prior Gram stain slides from laboratory 2 and identified one slide containing a questionable coryneform bacillus. The Gram stain had originated from the blood culture of laboratory worker 1. This blood culture had been drawn from laboratory worker 1 during her visit to the emergency room of laboratory worker 2's hospital, early in November 2001. Approximately 2 months before her illness, laboratory worker 2 had personally processed laboratory worker 1's blood culture but had characterized the isolate as coryneform bacilli. Wadsworth Center staff reviewed the original Gram stain from laboratory worker 1's blood culture specimen and noted gram-variable organisms with similar size and shape to *Brucella* organisms. No isolate was available for confirmation. However, laboratory worker 2 did not identify any other Gram stains that resembled *Brucella* organisms during the period before her illness.

The likely source of laboratory worker 1's infection was found after reassessing Gram stains performed in laboratory 1 in August, September, and October 2001. A 76-year-old woman visited the emergency room served by laboratory 1 in early September 2001. The patient had had fever (temperature 38.3ºC) for 1 day and headache for 3 weeks. She received one dose of ciprofloxacin for a possible urinary tract infection and was discharged. After 4 days of incubation, a Gram stain of this patient's blood culture specimen showed tiny, gram-negative coccobacilli, reported as coryneform bacilli. As part of the investigation for the source of infection for laboratory worker 1, this patient's prepared Gram stain slide was referred to the Wadsworth Center, where small, gram-variable cocci were identified. More than 1 year after being seen in the emergency room, this patient was offered *Brucella* SAT and a repeat blood culture. She refused *Brucella* SAT, and her physician did not identify symptoms consistent with brucellosis. Six months after this identification, further experimental analysis by the Wadsworth Center with polymerase chain reaction tests performed on Gram stain material detected *B. melitensis* DNA (Wadsworth Center, NYSDOH, unpub. data). The patient was again contacted, and *Brucella* SAT (>1.5 years after her initial blood culture) showed a titer of 1:80 (indeterminate). Repeat blood cultures were negative. Upon interview, the patient denied any visits outside the United States since 1989, when she emigrated from Peru. She also denied consuming any unpasteurized products from the United States or abroad. An additional interview was scheduled, but the patient died unexpectedly.

## Conclusions

Although brucellosis is a rare disease in the United States, its potential use as a bioweapon highlights the need for accurate and rapid identification (*15*). In this investigation, brucellosis was diagnosed weeks to years after initial positive blood cultures were misidentified, and laboratory personnel were unknowingly exposed to the organism.

This investigation suggests that transmission occurred from the 76-year-old index patient to laboratory worker 1, on the basis of *B. melitensis* DNA found in the Gram stain material. Processing the index patient's blood culture specimen on an open laboratory bench was most likely the reason for laboratory worker 1's illness approximately 5 weeks later. The same mechanism of transmission probably occurred when laboratory worker 2 handled laboratory worker 1's blood culture specimen on the open bench for biochemical testing. In these instances, blood culture bottle media were transferred to slides and agar without much risk for aerosolization, since contents were manipulated with a syringe. Biochemical tests, however, included the catalase test, which creates bubbles as a result of exposure to 3% hydrogen peroxide in positive specimens. *Brucella* spp. are known to be catalase positive. Neither laboratory worker could identify any other possible sources of infection, and because brucellosis is a rare disease in New York, the connection between these three patients is plausible. With the initial interpretations of these Gram stains as gram-variable, which resulted in misidentification of the organism by three different laboratories, NYSDOH initiated an effort to educate clinical laboratories in New York State about the potential difficulties in characterizing the organism and the importance of primary Gram stain interpretation. Additional investigation into the staining properties of *Brucella* spp. under various conditions is now in progress and may help differentiate *Brucella* spp. from other organisms.

Because of immigration and foreign travel, brucellosis remains an occupational hazard for laboratory personnel, even in industrialized countries where animal control efforts have virtually eliminated the disease. Because of the nonspecific symptoms and the rarity of the disease in the United States, healthcare providers may not consider brucellosis in a differential diagnosis. However, eliciting travel and occupational histories may assist in diagnosis. Moreover, improved communication among healthcare providers and laboratory personnel should facilitate prompt and accurate identification and appropriate handling of the organism. Its potential use as a bioweapon necessitates that healthcare providers, as well as microbiologists in hospitals and commercial laboratories, be knowledgeable about the diagnosis, identification, and handling of *Brucella* spp.

Reporting brucellosis cases to public health officials is another component in protecting others from this disease. Public health officials should notify laboratory personnel who may have handled cultures taken from patients ultimately diagnosed with brucellosis. Early notification of exposed personnel could lead to their timely diagnosis and treatment, should symptoms occur, and could prevent further laboratory exposures.
